# Altered localization of nucleoporin 98 in primary tauopathies

**DOI:** 10.1093/braincomms/fcac334

**Published:** 2022-12-22

**Authors:** John R Dickson, Matthew P Frosch, Bradley T Hyman

**Affiliations:** Department of Neurology, MassGeneral Institute for Neurodegenerative Disease, Massachusetts General Hospital, Charlestown, MA, USA; Harvard Medical School, Boston, MA, USA; Harvard Medical School, Boston, MA, USA; Department of Pathology and Neurology Service, C.S. Kubik Laboratory for Neuropathology, Massachusetts General Hospital, Boston, MA, USA; Department of Neurology, MassGeneral Institute for Neurodegenerative Disease, Massachusetts General Hospital, Charlestown, MA, USA; Harvard Medical School, Boston, MA, USA

**Keywords:** mislocalization, nucleoporin, NUP98, tau, tauopathy

## Abstract

Nucleoporin 98 is a nuclear pore complex component that is mislocalized in Alzheimer’s disease and the alteration in nucleoporin 98 has been attributed to tau. In order to determine if nucleoporin 98 mislocalization is a general feature of tauopathies, we assessed the localization of nucleoporin 98 in neurons in primary tauopathies, including frontotemporal lobar degeneration-tau, corticobasal degeneration and progressive supranuclear palsy. Immunofluorescence staining was performed on frontal cortex and occipital cortex tissue from cases of primary tauopathies and controls without neurodegenerative disease using antibodies to identify nucleoporin 98, phospho-tau (Ser202, Thr205) monoclonal antibody and neuronal marker microtubule-associated protein 2. The stained tissue was imaged by fluorescence microscopy and the number of neurons with mislocalized nucleoporin 98 and phospho-tau (Ser202, Thr205) monoclonal antibody staining was quantified. In frontal cortex tissue, all primary tauopathies examined demonstrated significantly increased numbers of neurons with abnormal localization of nucleoporin 98 along the nuclear envelope compared with control tissue. Additionally, frontotemporal lobar degeneration-tau and corticobasal degeneration in the frontal cortex demonstrated significantly increased numbers of neurons with a cytoplasmic mislocalization of nucleoporin 98 compared with control tissue. The number of neurons with mislocalized nucleoporin 98 was significantly correlated with the number of neurons with phospho-tau (Ser202, Thr205) monoclonal antibody-positive tau staining. In the occipital cortex, which is relatively spared from pathological tau accumulations in these primary tauopathies, the localization of nucleoporin 98 was not significantly altered. This study demonstrates that nucleoporin 98 mislocalization is a feature of primary tauopathies and is associated with pathological tau accumulation. In the context of prior research demonstrating nucleoporin 98 mislocalization in Alzheimer’s disease and an interaction between tau and nucleoporin 98, these results further support the hypothesis that pathological tau may contribute to nucleoporin 98 mislocalization. Given the critical role of the nuclear pore complex in nucleocytoplasmic transport, the identification of nucleoporin 98 mislocalization in primary tauopathies highlights a potential pathophysiological disruption in these disorders.

## Teaser

Dickson *et al*. report that the nuclear pore complex component nucleoporin 98 is mislocalized in primary tauopathies and that the degree of nucleoporin 98 mislocalization is correlated with the burden of pathological tau aggregates. These results indicate nucleoporin 98 mislocalization is a feature of primary tauopathies.

## Graphical Abstract

**Graphical abstract:**
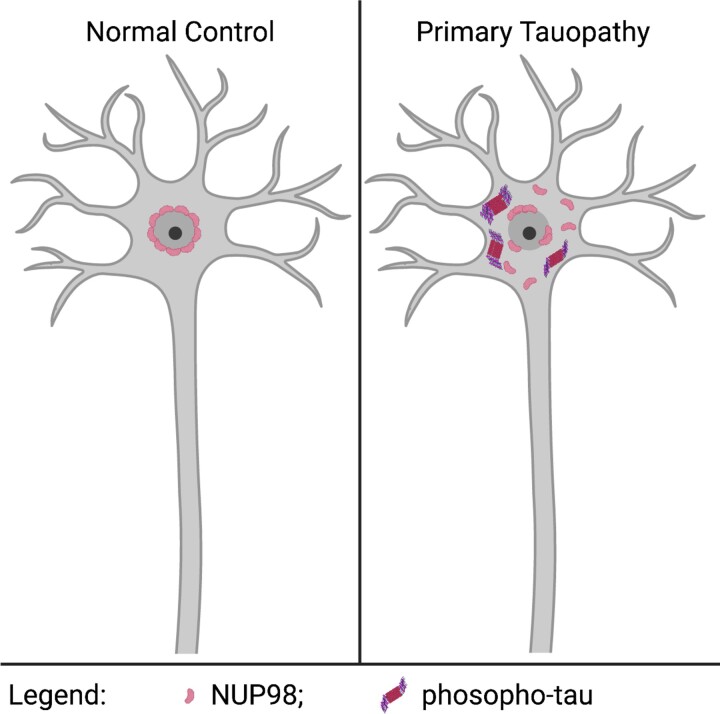

## Introduction

The microtubule-associated protein tau (encoded by the *MAPT* gene^1^) accumulates in a variety of neurodegenerative diseases known as tauopathies,^2^ including frontotemporal lobar degeneration-tau (FTLD-tau),^3^ corticobasal degeneration (CBD),^4^ progressive supranuclear palsy (PSP)^5^ and Alzheimer’s disease.^6^ Despite the clinical and neuropathological heterogeneity of these disorders, the primary phenotype of intraneuronal tau misfolding and accumulation suggests that shared pathophysiological mechanisms related to neurodegeneration are possible.^2^ Our laboratory has previously shown that in Alzheimer’s disease, tau interacts with nucleoporin 98 (NUP98), a component of the nuclear pore complex and that NUP98 is mislocalized away from the nuclear membrane to the cytoplasm.^7^ Furthermore, this study demonstrated that reducing expression of the frontotemporal dementia-related pathogenic P301L^8-11^ tau within a mouse model of tauopathy^12^ rescued NUP98 mislocalization and nucleocytoplasmic transport defects.^7^ These observations raise the question of whether NUP98 mislocalization could be a general feature of tauopathies.

In order to test the hypothesis that NUP98 mislocalization occurs in primary tauopathies, we examined the localization of NUP98 in FTLD-tau, CBD and PSP. We found that NUP98 mislocalization occurs in the frontal cortex of primary tauopathies and is correlated with the burden of pathological tau. In contrast, the primary visual cortex, which is largely spared from pathological changes in these diseases, did not demonstrate substantial NUP98 mislocalization. Overall, these data suggest that the nuclear pore complex component NUP98 is mislocalized in several primary tauopathies, supporting the hypothesis that misfolded tau induces nuclear pore complex disruption across multiple tauopathies.

## Materials and methods

### Cases

Cases with a neuropathological diagnosis of FTLD-tau,^3^ CBD^4^ and PSP^5^ were identified in the Massachusetts Alzheimer’s Disease Research Center brain bank. Given the prior results in a mouse model of tauopathy expressing the P301L *MAPT* mutation,^7^ the three available FTLD-tau cases with the P301L *MAPT* mutation were studied. Cases without substantial neuropathological findings were used as controls (Table 1). Formalin-fixed, paraffin-embedded (FFPE) regions of the frontal cortex (Brodmann areas 8,9) and occipital cortex (Brodmann area 17) of the cases and controls were identified in the brain bank. In some cases, either frontal or occipital tissue was not available for the selected cases, as indicated in Table 1. In cases where the tissue was unavailable, the samples with unavailable tissue were omitted from the analysis. The FFPE tissue was sectioned on an RM 2155 microtome (Leica Microsystems Inc., Buffalo Grove, IL, USA) at a thickness of 7 µm and mounted on Fisherbrand Superfrost Plus Microscope Slides (Thermo Fisher Scientific, Waltham, MA, USA).

**Table 1:** Case characteristics

Arbitrary case #	Diagnosis	Age	Sex	PMI	Notes
1	Control	47	Male	3	–
2	Control	60	Male	11	–
3	Control	68	Female	20	–
4	Control	76	Female	48	–
5	Control	89	Female	8	–
6	Control	90+	Male	23	–
7	FTLD-tau	33	Male	33	P301L
8	FTLD-tau	56	Male	26	P301L; occipital tissue unavailable
9	FTLD-tau	70	Male	12	P301L
10	FTLD-tau	71	Female	4	Poor occipital staining
11	FTLD-tau	71	Female	72	–
12	FTLD-tau	98	Female	NA	–
13	CBD	64	Male	18	Frontal tissue unavailable
14	CBD	67	Female	17	–
15	CBD	71	Male	18	–
16	CBD	74	Male	18	–
17	CBD	80	Male	12	–
18	PSP	49	Male	24	Poor occipital staining
19	PSP	79	Male	4	–
20	PSP	79	Male	10	–
21	PSP	81	Female	16	–
22	PSP	94	Female	24	–

CBD, corticobasal degeneration; FTLD-tau, frontotemporal lobar degeneration-tau; NA, not available; PMI, post-mortem interval (in hours); PSP, progressive supranuclear palsy.

### Immunofluorescence staining

Immunofluorescence staining was performed generally as previously described.^13^ In brief, the FFPE sections were prepared for immunofluorescence staining by deparaffinization in xylenes (×2, 5 min each) and rehydration through a series of ethanol grades to water (100% ethanol × 2, 95% ethanol, 70% ethanol, 50% ethanol, deionized water × 2, 5 min each). Antigen retrieval was performed in a humidified chamber for 1 h at 95–100°C using an IHC-Tek epitope retrieval solution (IHCworld, Woodstock, MD, USA). The slides were cooled on ice for 10 min and then permeabilized for 8 min using 0.4% Triton X-100 (Millipore Sigma, Burlington, MA, USA) in phosphate-buffered saline. Blocking was performed for 1.5 h at room temperature with 10% normal goat serum in phosphate-buffered saline. The slides were incubated with primary antibodies (Table 2) diluted in antibody diluent (Abcam, Cambridge, UK) overnight at 4°C. The slides were washed with 5% normal goat serum with 0.5% Triton X-100 in Tris-buffered saline (×4, 2 min each). The slides were then incubated with secondary antibodies (Table 2) diluted in antibody diluent for 1.5 h at room temperature. The slides were washed with 5% normal goat serum with 0.5% Triton X-100 in Tris-buffered saline (×3, 2 min each) and then Tris-buffered saline (×2, 2 min each). Nuclear counterstain was performed with 4′,6-diamidino-2-phenylindole (DAPI; Millipore Sigma) diluted 1:1000 in phosphate-buffered saline with a 5 min incubation. Excess DAPI was removed by three 5 min washes with phosphate-buffered saline. To quench tissue autofluorescence, the slides were incubated in a 0.1% Sudan Black (Millipore Sigma) solution in 70% ethanol for 30 min. Excess Sudan Black was removed by three 5 min washes with phosphate-buffered saline. The slide was then prepared for mounting with Fluoromount G and DAPI (Southern Biotech, Birmingham, AL, USA). Gold Seal Cover Glass (Thermo Fisher Scientific) was placed on the slide and sealed with clear nail polish (Electron Microscopy Sciences, Hatfield, PA, USA).

**Table 2:** Antibodies

Antibody	Host	Manufacturer	Catalogue #	Dilution
NUP98	Rat	Abcam, Cambridge, UK	ab50610	1:200
Phospho-tau (Ser202, Thr205) (AT8)	Mouse	Thermo Fisher Scientific, Waltham, MA, USA	MN1020	1:200
MAP2	Chicken	Abcam, Cambridge, UK	ab5392	1:400
Anti-rat IgG H&L (Alexa Fluor 555)	Goat	Abcam, Cambridge, UK	ab150166	1:200
Anti-mouse IgG (H + L), Alexa Fluor 488	Goat	Thermo Fisher Scientific, Waltham, MA, USA	A28175	1:200
Anti-chicken IgY (H + L), Alexa Fluor 647	Goat	Thermo Fisher Scientific, Waltham, MA, USA	A32933	1:200

### Microscopy

Slides were imaged using a FLUOVIEW FV3000 Confocal Laser Scanning Microscope (Olympus, Tokyo, Japan) and images used for publication were acquired with a 60× oil objective (numerical aperture 1.30). The confocal images were visualized using Fiji.^14^ The panels presented for publication were prepared using the EZFig plugin.^15^ For quantitative analysis, the slides were digitized using a VS120 Virtual Slide Microscope (Olympus) with a 40× objective (numerical aperture 0.95).

### Quantification

A 0.25 µm × 0.25 µm grid was overlaid on the digitized slides using cellSens software (Olympus). For each slide, two different cortical regions were identified for quantification with the following criteria: the orientation of the cortex on the slide was roughly horizontal or vertically relative to the screen and all six cortical layers were present without artefactual disruption of the tissue architecture. For each region, the 0.25 µm × 0.25 µm (0.0625 µm2) squares in the grid for quantification were chosen by selecting a square containing Layer I of the cortex and then selecting successive diagonal squares through to the subcortical white matter adjacent to Layer VI, then selecting the successive diagonal squares back up to Layer I of the cortex, effectively forming a ‘V’ shape. Using this method, an average of 14.3 squares (median 14, mode 18, range 9–18) per case were utilized for quantification in the frontal cortex and an average of 13.2 squares (median 13, mode 15, range 10–17) per case were utilized for quantification in the occipital cortex. Neurons were identified by microtubule-associated protein 2 (MAP2) staining and nuclei were identified by DAPI staining. For each square, a neuron would be considered for quantification if its nucleus was fully present within the square. The following metrics were quantified for each square: number of MAP2-positive neurons, number of neurons with abnormal distribution of NUP98 along the nuclear envelope, number of neurons with NUP98 present in the cytoplasm and number of neurons staining positive for phospho-tau with the phospho-tau (Ser202, Thr205) monoclonal (AT8) antibody. The percentage of neurons with abnormal nuclear NUP98 localization was calculated as the number of neurons with abnormal distribution NUP98 along the nuclear envelope divided by the number of MAP2-positive neurons times 100%. The percentage of neurons with abnormal cytoplasmic NUP98 localization was calculated as the number of neurons with NUP98 present in the cytoplasm divided by the number of MAP2-positive neurons times 100%. The samples were identified by a number corresponding to the order in which the case was added to the brain bank. Since the tau staining pattern with the AT8 antibody could reveal information about a likely diagnosis, the channel corresponding to this antibody was counted last in order to limit potential bias when performing quantification with the other channels.

Given the differences in tissue availability and quality of this convenience sample (Table 1), the sample size of the cases used for quantification was different between frontal cortex and occipital cortex samples. For the frontal cortex, six control, six FTLD-tau, four CBD and five PSP samples were quantified. For the occipital cortex, six control, four FTLD-tau, five CBD and four PSP samples were quantified.

### Statistical analysis

The statistical analysis was performed using Prism 9.3.1 software (GraphPad, San Diego, CA, USA), except where noted. Comparisons among multiple groups were performed with a Kruskal–Wallis test and an adjustment for multiple comparisons with Dunn’s multiple comparisons test. Comparisons between two groups were performed with a Mann–Whitney U test. Correlation analysis was performed with the Pearson *r* test or Spearman correlation, as indicated. A simple linear regression was used to visualize the trend of the correlational data. A Fisher’s exact test was used to evaluate categorical data and was performed using R.^16^ Statistical significance was determined at an α-level of <0.05.

## Results

**Figure 1:**
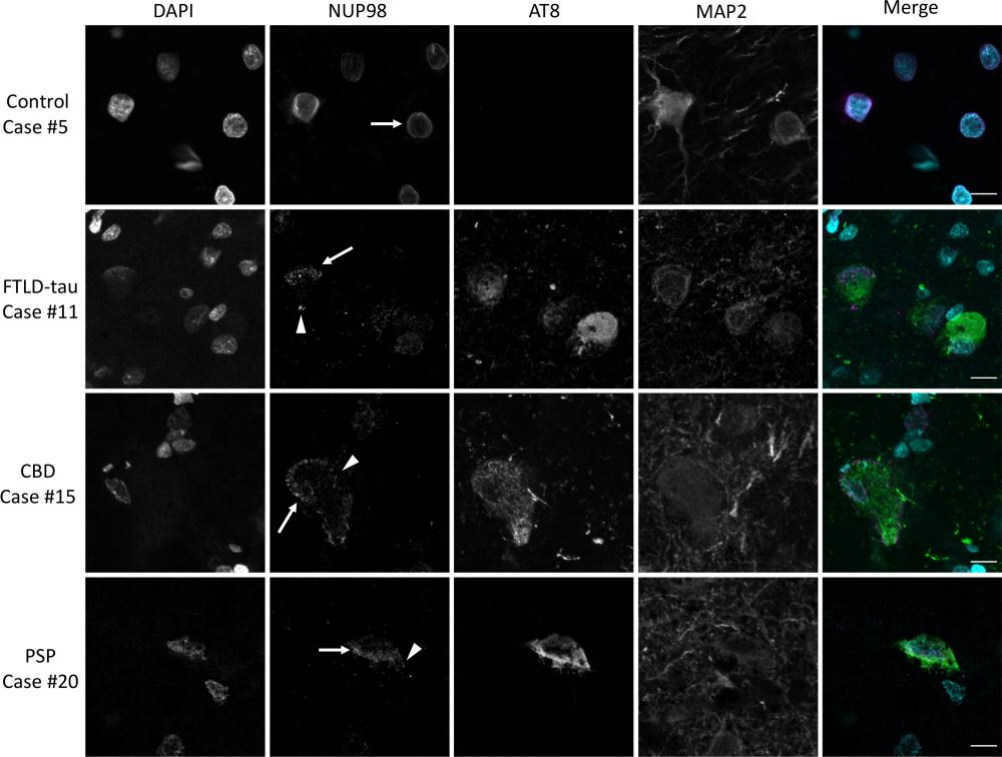
**Mislocalization of NUP98 in primary tauopathy frontal cortex.** Frontal cortex (Brodmann areas 8 and 9) sections from neurologically normal controls, FTLD-tau, CBD, or PSP cases were stained for NUP98, phospho-tau (AT8) and MAP2 by immunofluorescence and nuclei were counterstained with DAPI. Merged images demonstrate DAPI in cyan, NUP98 in magenta and AT8 in green. MAP2 is omitted from the merged images for the sake of clarity. Arrow demonstrates nuclear NUP98 staining. Arrowhead demonstrates cytoplasmic NUP98 staining. Scale bar = 10 µm.

The NUP98 staining pattern in frontal cortex tissue was examined by confocal microscopy and representative images are presented to demonstrate the findings. The NUP98 staining pattern in control frontal cortex neurons generally demonstrated the expected uniform distribution along the nuclear envelope (Fig. 1). In contrast, some neurons within the frontal cortex of primary tauopathy cases of FTLD-tau, CBD and PSP showed abnormal localization of NUP98 (Fig. 1). The abnormal NUP98 localization was apparent in two distinct ways: (i) abnormal, non-uniform distribution along the nuclear envelope and (ii) abnormal mislocalization to the cytoplasm (Fig. 1). The abnormal localization of NUP98 was often, though not always, present in neurons that also stained positive for the pathology-associated phospho-tau antibody AT8 (Fig. 1).

**Figure 2:**
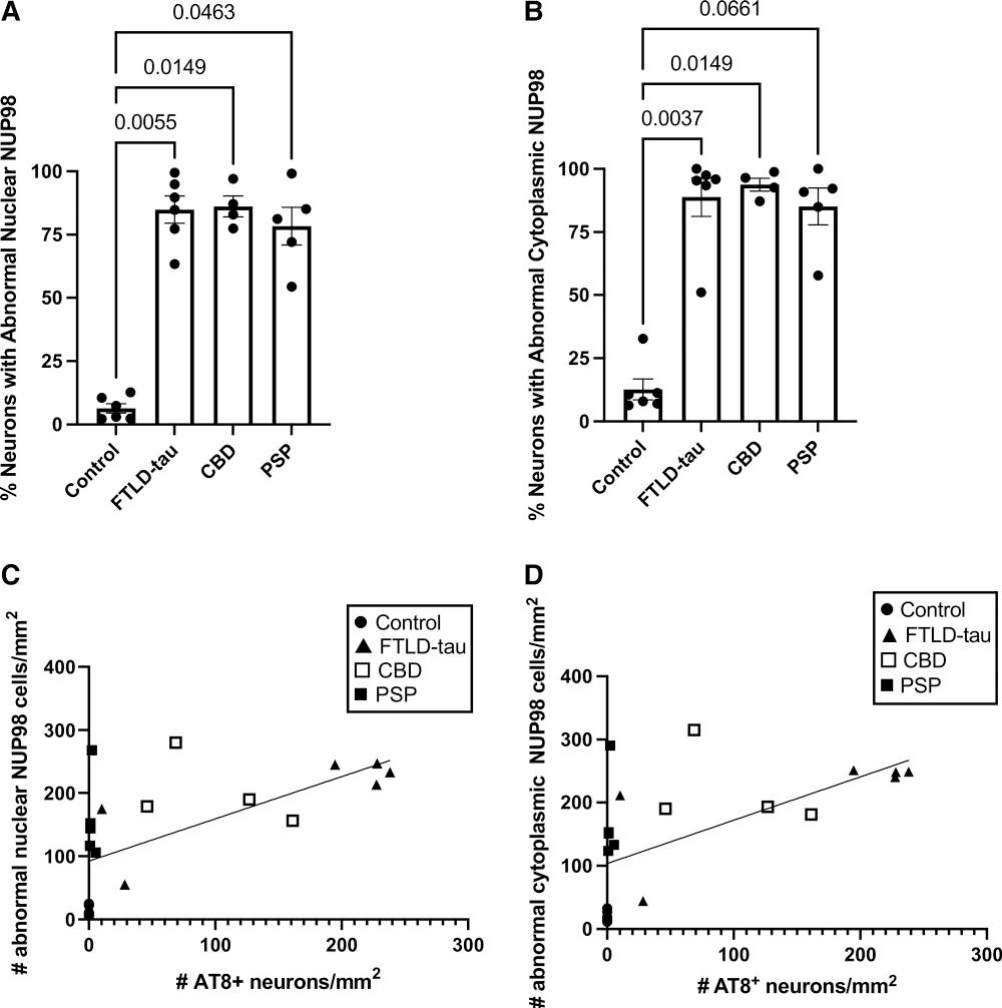
**Quantitative evaluation of NUP98 mislocalization in the frontal cortex of primary tauopathies.** The mislocalization of NUP98 in MAP2-positive neurons from the frontal cortex (Brodmann areas 8 and 9) of control (*n* = 6), FTLD-tau (*n* = 6), CBD (*n* = 4) and PSP (*n* = 5) cases was determined using stereology-based quantification using a grid with 0.25 µm × 0.25 µm squares. The number of MAP2-positive neurons with AT8-positive tau staining was quantified using the same methodology. (**A**) Percentage of MAP2-positive neurons with abnormal localization of NUP98 along the nuclear envelope. Statistical analysis was performed with the Kruskal–Wallis test and Dunn’s multiple comparison test. Adjusted *P*-values are indicated. (**B**) Percentage of MAP2-positive neurons with abnormal cytoplasmic localization of NUP98. Statistical analysis was performed with the Kruskal–Wallis test and Dunn’s multiple comparison test. Adjusted *P*-values are indicated. (**C**) Correlation of the number of MAP2-positive neurons with abnormal nuclear NUP98 localization with the number of MAP2-positive neurons with AT8-positive tau staining. The line represents the best fit of simple linear regression. Pearson’s *r* = 0.6279, *P* = 0.0023. (**D**) Correlation of the number of MAP2-positive neurons with abnormal cytoplasmic NUP98 localization with the number of MAP2-positive neurons with AT8-positive tau staining. The line represents the best fit of simple linear regression. Pearson’s *r* = 0.6065, *P* = 0.00036.

In order to examine these findings quantitatively, we used virtual slide images obtained using a VS120 Virtual Slide Microscope (example images shown in Supplementary Fig. 1) to perform a stereology-informed approach to count the number of neurons with abnormal NUP98 localization. We also quantified the number of neurons containing AT8-positive phospho-tau. The percentage of frontal cortex neurons with abnormal nuclear localization of NUP98 was significantly higher for FTLD-tau, CBD and PSP compared with control cases (Fig. 2A). The percentages of frontal cortex neurons with abnormal cytoplasmic localization of NUP98 were significantly higher in FTLD-tau and CBD compared with controls (Fig. 2B). For PSP cases, there was a trend towards an increased percentage of neurons with abnormal cytoplasmic localization of NUP98, though this trend failed to reach statistical significance (Fig. 2B). Given the observation that NUP98 mislocalization was often present in AT8-positive neurons, we performed a correlation analysis of a normal nuclear NUP98 localization (Fig. 2C) or abnormal cytoplasmic NUP98 localization (Fig. 2D) with AT8 staining for all cases using a Pearson *r*. The number of AT8-positive neurons is significantly correlated with both the number of neurons with abnormal nuclear NUP98 localization (Fig. 2C, *P* = 0.0062) and the number of neurons with abnormal cytoplasmic NUP98 localization (Fig. 2D, *P* = 0.0076).

**Figure 3:**
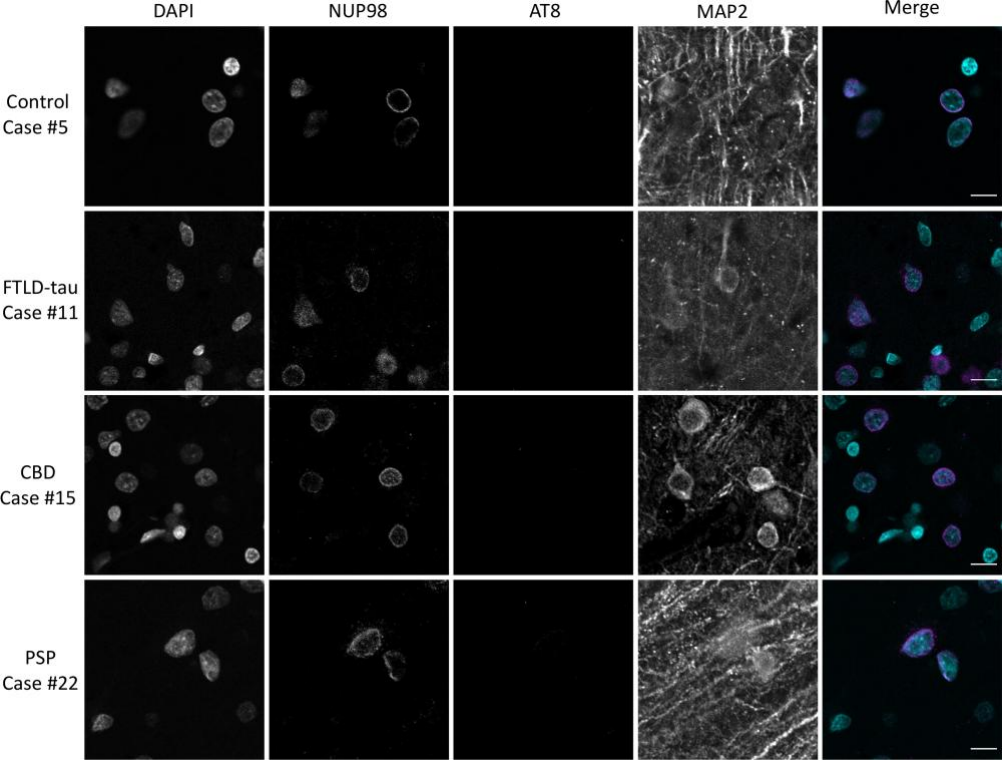
**Localization of NUP98 in primary tauopathy occipital cortex.** Calcarine cortex (Brodmann area 17) sections from cognitively normal controls, FTLD-tau, CBD, or PSP cases were stained for NUP98, phospho-tau (AT8) and MAP2 by immunofluorescence and nuclei were counterstained with DAPI. Merged images demonstrate DAPI in cyan, NUP98 in magenta and AT8 in green. MAP2 is omitted from the merged images for the sake of clarity. Scale bar = 10 µm.

To evaluate whether these changes in NUP98 localization are generally present in neurons in primary tauopathies or whether these changes are present in areas that tend to be pathologically spared in these diseases, we performed immunohistochemical stainings with NUP98, AT8 and MAP2 antibodies in primary visual cortex from the same set of cases. However, the occipital cortex tissue from three cases was excluded from further analysis due to lack of tissue (Arbitrary case number 8, Table 1) or poor tissue staining with a high background in all channels (Arbitrary case numbers 10 and 18, Table 1). The pattern of NUP98 staining was evaluated by confocal microscopy and representative images are presented to demonstrate the findings. Immunohistochemical staining of the primary visual cortex with NUP98 revealed the expected uniform staining of the nuclear envelope without cytoplasmic mislocalization in most neurons in both control and primary tauopathy (FTLD-tau, CBD and PSP) cases (Fig. 3). In general, AT8 staining was largely absent in neurons throughout the primary visual cortex (Fig. 3). The only exception to this was in the two cases of FTLD-tau with the pathogenic tau P301L mutation for which occipital cortex tissue was available (Arbitrary case numbers 7 and 9), which did show AT8 staining in some neurons in the primary visual cortex (an example image is shown in Supplementary Fig. 2).

**Figure 4:**
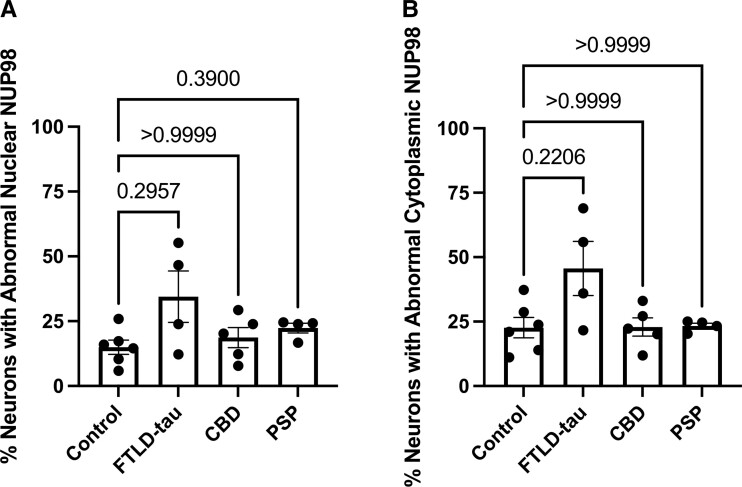
**Quantitative evaluation of NUP98 mislocalization in occipital cortex of primary tauopathies.** The mislocalization of NUP98 in MAP2-positive neurons from occipital cortex (Brodmann area 17) of control (*n* = 6), FTLD-tau (*n* = 4), CBD (*n* = 5) and PSP (*n* = 4) cases was determined using stereology-based quantification us a grid with 0.25 µm × 0.25 µm squares. The number of MAP2-positive neurons with AT8-positive tau staining was quantified using the same methodology. (**A**) Percentage of MAP2-positive neurons with abnormal localization of NUP98 along the nuclear envelope. Statistical analysis was performed with the Kruskal–Wallis test and Dunn’s multiple comparison test. Adjusted *P*-values are indicated. (**B**) Percentage of MAP2-positive neurons with abnormal cytoplasmic localization of NUP98. Statistical analysis was performed with the Kruskal–Wallis test and Dunn’s multiple comparison test. Adjusted *P*-values are indicated.

A stereology-informed approach with virtual slide images (an example shown in Supplementary Fig. 2) was used to quantify abnormal NUP98 localization in neurons in the primary visual cortex. There was no significant difference between any of the primary tauopathy cases and neurologically normal controls for both abnormal NUP98 nuclear localization (Fig. 4A) and abnormal NUP98 cytoplasmic localization (Fig. 4B). Of note, the two cases with the highest percentages of abnormal NUP98 localization were the two cases of FTLD-tau with the pathogenic P301L mutation. Since the vast majority of specimens lacked AT8 staining in the primary visual cortex, no correlational analysis with AT8 and NUP98 staining patterns were performed for the primary visual cortex.

In order to evaluate variables of age at death, post-mortem interval and sex as potential confounders, each variable was examined for a relationship with the outcomes of interest in our analysis (percentage neurons with abnormal localization of NUP98). Additionally, these variables were also evaluated for their relationship with the diagnoses included in this study. Statistical analysis of these relationships did not reveal any statistically significant relationship between the variables of age at death, post-mortem interval, or sex and the outcomes of interest (Supplementary Table 1), suggesting that these variables are unlikely to be significant confounders in the analyses presented above.

## Discussion

The mislocalization of NUP98 along the nuclear envelope and in the cytoplasm in the frontal cortex of primary tauopathies (Fig. 1) is consistent with the previous similar observations in Alzheimer’s disease.^7^ The altered localization of NUP98 along the nuclear envelope leading to a non-uniform distribution of NUP98 around the nucleus is seen in FTLD-tau, CBD and PSP (Fig. 2A). The cytoplasmic mislocalization of NUP98 was statistically distinct from controls in FTLD-tau and CBD (Fig. 2B), with PSP showing a non-significant trend towards increased cytoplasmic NUP98 mislocalization (Fig. 2B). Overall, these data combined with prior work in Alzheimer’s disease^7^ indicate that NUP98 mislocalization is a general feature of tauopathies, including primary and secondary tauopathies as well as 3R, 4R and 3R/4R tauopathies.

The correlations between AT8-positive pathological tau burden and nuclear envelope (Fig. 2C) or cytoplasmic (Fig. 2D) mislocalization of NUP98, combined with the general sparing of primary visual cortex from NUP98 mislocalization in addition to AT8-positive tau accumulation (Figs 3 and 4), are consistent with the hypothesis that pathological tau has a relationship to NUP98 mislocalization. The demonstration of correlation rather than causation is a limitation of this study. Of note, the limited sample size of each category also limits inferences about any potential differences among tauopathies. The impact of pathological *MAPT* mutations on nuclear pore complex localization and nucleocytoplasmic transport in animal and cellular models of tauopathy^7,17^ also suggests a causal relationship between pathological tau and nuclear pore complex disruption. Taken together, this study along with previously published reports suggests that the presence of pathological tau impacts nuclear pore complex and specifically NUP98, localization.

The present study does not assess the mechanism by which NUP98 mislocalization occurs in primary tauopathies. However, one possible cause of the nuclear envelope and/or cytoplasmic mislocalization is an interaction with pathological tau aggregates, as a direct interaction between tau and NUP98 has been demonstrated previously.^7^ Another possible cause of NUP98 mislocalization is a microtubule-induced deformation of the nucleus, which causes invaginations of the nuclear envelope and altered nucleocytoplasmic transport in frontotemporal dementia caused by a *MAPT* mutation.^17^ Either mechanism raises the possibility that nucleocytoplasmic transport, a highly regulated cell function key to environmental responses, may be disrupted in tauopathies.

In addition to the observations in Alzheimer’s disease,^7^ disruptions in the nuclear pore complex and nucleocytoplasmic transport have been previously described in several neurodegenerative diseases. The pathogenic hexanucleotide repeat expansion of Chromosome 9 open reading frame 72 in amyotrophic lateral sclerosis and frontotemporal-spectrum disease has been associated with alterations in nuclear pore complex structure and nucleocytoplasmic transport defects.^18,19^ Similarly, pathogenic mutations in fused sarcoma causing amyotrophic lateral sclerosis have also been shown to disrupt the nuclear pore complex and nucleocytoplasmic transport.^20^ The CAG repeat expansion in the *Huntingtin* gene leads to the mislocalization of nuclear pore complex components and defective nucleocytoplasmic transport.^21^ The polyglutamine expansion in the *ataxin-1* gene that causes spinocerebellar ataxia Type 1 has been implicated in alterations in nuclear pore complex components, including NUP98, and nucleocytoplasmic transport.^22^ These observations suggest that disruption of the nuclear pore complex is a common feature of many neurodegenerative diseases.

The present study focused on neuronal NUP98 mislocalization in primary tauopathies. However, pathological tau accumulations are also found in glial cells in certain primary tauopathies. For example, neuropathological findings in PSP include tau-positive tufted astrocytes and tau-positive coiled bodies and threads in oligodendrocytes.^23^ Oligodendroglial coiled bodies and threads are also found in CBD.^23^ In this context, future studies investigating glial nuclear pore complex mislocalization in primary tauopathies are warranted.

In summary, our findings that NUP98 mislocalization occurs in neurons in the frontal cortex of FTLD-tau, CBD and PSP cases reveal NUP98 as a feature of primary tauopathies. The number of neurons with NUP98 mislocalization is significantly correlated with the burden of AT8-positive phospho-tau, raising the possibility that pathological tau could contribute to the NUP98 mislocalization. Overall, our study adds to the growing body of literature implicating nuclear pore complex disruption in the pathophysiology of several neurodegenerative diseases.

## Supplementary Material

fcac334_Supplementary_Data

## Data Availability

The data supporting the findings of this study will be made available upon reasonable request to the corresponding author.

## Abbreviations

AT8 =: phospho-tau (Ser202, Thr205) monoclonal antibody
CBD =: corticobasal degeneration
DAPI =: 4′,6-diamidino-2-phenylindole
FFPE =: formalin-fixed, paraffin-embedded
FTLD-tau =: frontotemporal lobar degeneration-tau
NUP98 =: nucleoporin 98
PSP =: progressive supranuclear palsy
