# Supplementary material for: Altered localization of nucleoporin 98 in primary tauopathies
Source: Brain Commun. 2022 Dec 22;5(1):fcac334. doi: 10.1093/braincomms/fcac334 (PMC9806717; doi:10.1093/braincomms/fcac334)
Supplement: fcac334_Supplementary_Data [file fcac334_supplementary_data.pdf]

Altered Localization of Nucleoporin 98 in Primary Tauopathies

Supplementary Data

Supplementary Table 1. Evaluation of Potential Confounding Variables.

**Variable**	**Brain Region**	**Outcome**	**Statistical Test**	***p*-value**
Age at death	Frontal cortex	% Abnormal nuclear NUP98	Spearman correlation	0.1747
Age at death	Frontal cortex	% Abnormal cytoplasmic NUP98	Spearman correlation	0.4242
Age at death	Occipital cortex	% Abnormal nuclear NUP98	Spearman correlation	00.6702
Age at death	Occipital cortex	% Abnormal cytoplasmic NUP98	Spearman correlation	0.5180
Age at death	NA	Diagnosis	Kruskal-Wallis test	0.6571
PMI	Frontal cortex	% Abnormal nuclear NUP98	Spearman correlation	0.9623
PMI	Frontal cortex	% Abnormal cytoplasmic NUP98	Spearman correlation	0.7691
PMI	Occipital cortex	% Abnormal nuclear NUP98	Spearman correlation	0.5386
PMI	Occipital cortex	% Abnormal cytoplasmic NUP98	Spearman correlation	0.3460
PMI	NA	Diagnosis	Kruskal-Wallis test	0.7511
Sex	Frontal cortex	% Abnormal nuclear NUP98	Mann-Whitney U test	0.3451
Sex	Frontal cortex	% Abnormal cytoplasmic NUP98	Mann-Whitney U test	0.2251
Sex	Occipital cortex	% Abnormal nuclear NUP98	Mann-Whitney U test	0.7780
Sex	Occipital cortex	% Abnormal cytoplasmic NUP98	Mann-Whitney U test	0.3950
Sex	NA	Diagnosis	Fisher's exact test	0.7889

PMI = Post-mortem interval

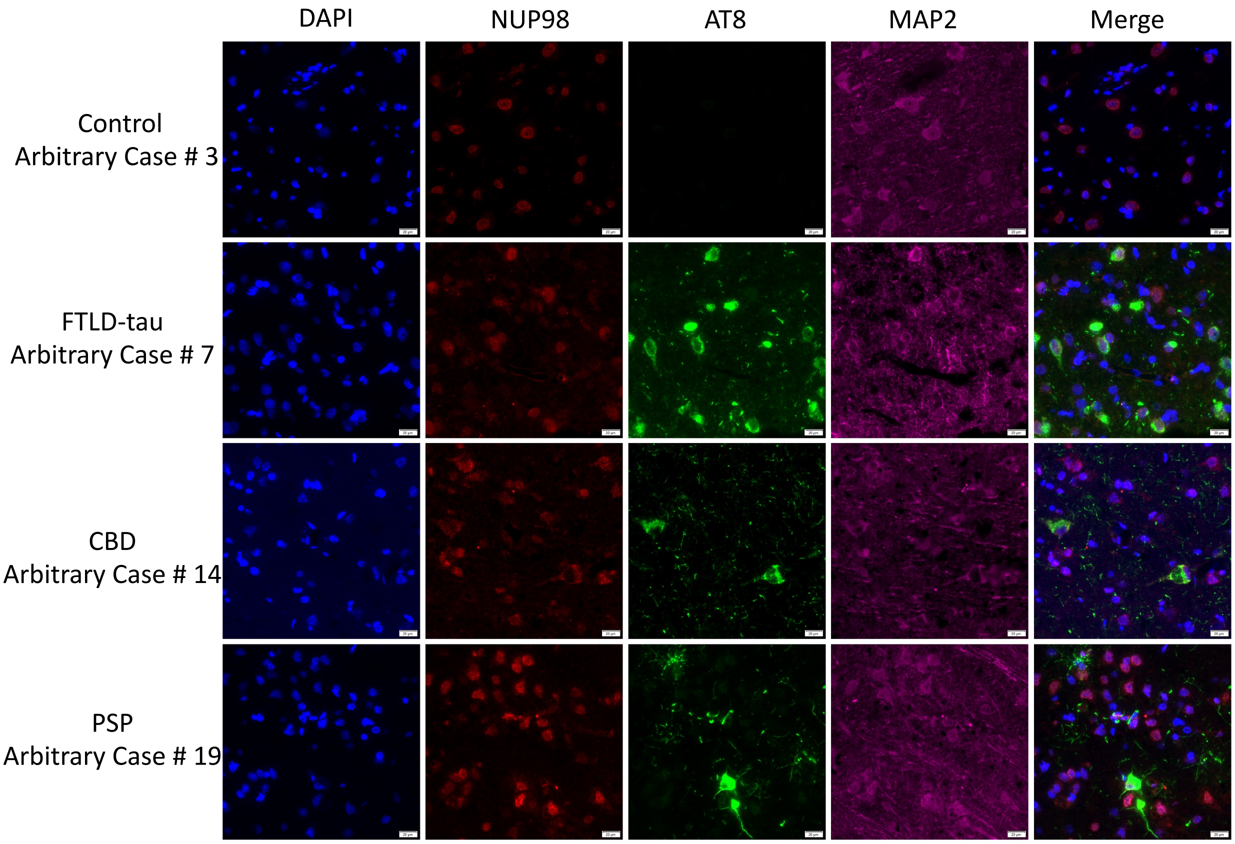

Supplementary Figure 1. Virtual Slide Images of NUP98 in Primary Tauopathy Frontal Cortex. Frontal cortex (Brodmann area 8,9) sections from neurologically normal controls, FTLD-tau, CBD, or PSP cases were stained for NUP98, phospho-tau (AT8), and MAP2 by immunofluorescence and nuclei counterstained with DAPI. The slides were imaged on the VS120 Virtual Slide Microscope. The arbitrary case numbers are indicated. Merged images demonstrate DAPI in blue, NUP98 in red, and AT8 in green. MAP2 is omitted from the merged images for the sake of clarity. Scale bar = 20 μm.

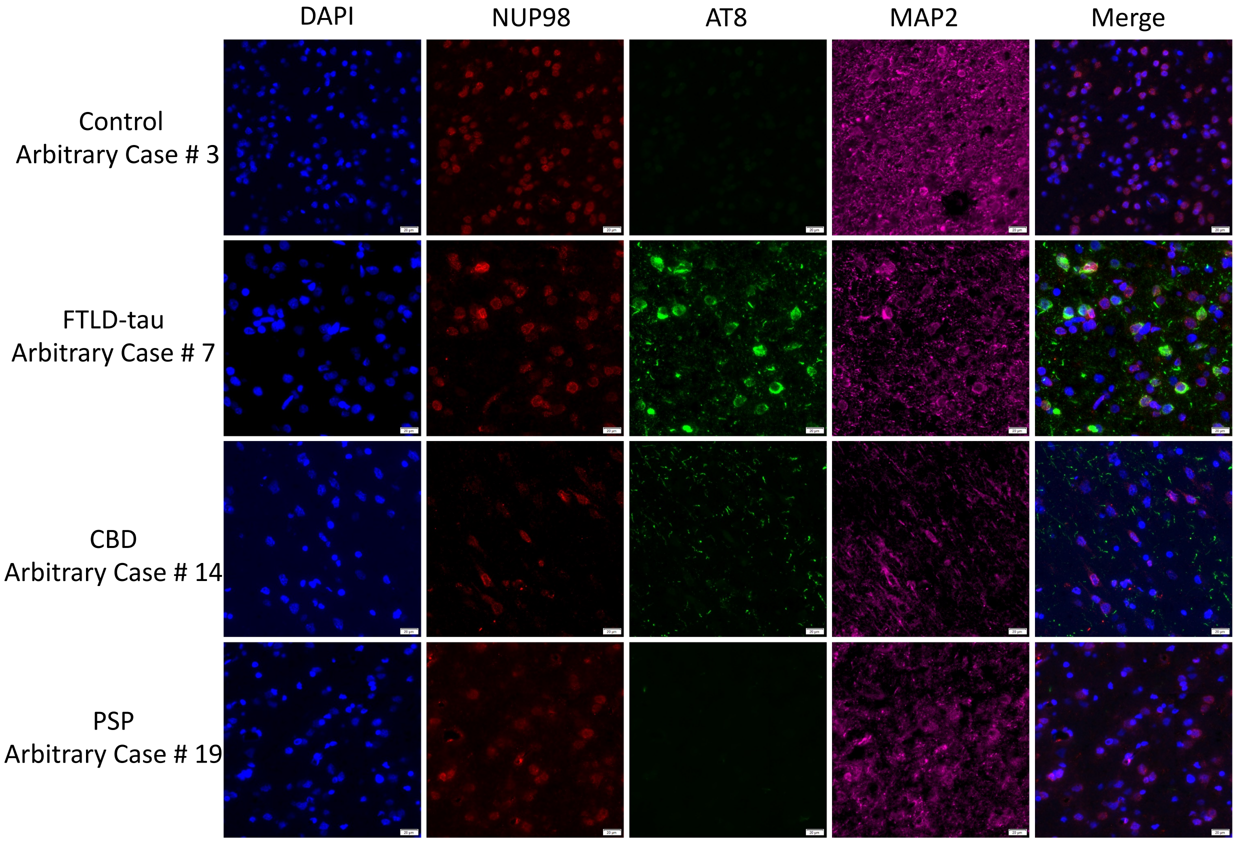

Supplementary Figure 2. Virtual Slide Images of NUP98 in Primary Tauopathy Occipital Cortex. Occipital cortex (Brodmann area 17) sections from neurologically normal controls, FTLD-tau, CBD, or PSP cases were stained for NUP98, phospho-tau (AT8), and MAP2 by immunofluorescence and nuclei counterstained with DAPI. The slides were imaged on the VS120 Virtual Slide Microscope. The arbitrary case numbers are indicated. Merged images demonstrate DAPI in blue, NUP98 in red, and AT8 in green. MAP2 is omitted from the merged images for the sake of clarity. Scale bar = 20 μm.
